# Overview of Molecular Detection Technologies for *MET* in Lung Cancer

**DOI:** 10.3390/cancers15112932

**Published:** 2023-05-26

**Authors:** Carina Heydt, Michaela Angelika Ihle, Sabine Merkelbach-Bruse

**Affiliations:** Faculty of Medicine, Institute of Pathology, University Hospital Cologne, University of Cologne, Kerpener Str. 62, 50937 Cologne, Germany

**Keywords:** MET, NSCLC, *MET* exon 14 skipping mutation, *MET* gene amplification, *MET* fusion

## Abstract

**Simple Summary:**

A variety of MET aberrations that lead to the dysregulation of the *MET* oncogene and thus the activation of various signaling pathways have been described. These include MET overexpression, the activation of *MET* mutations comprising exon 14 skipping mutations, *MET* gene amplifications, and *MET* fusions. Patients with such aberrations can be treated using a targeted inhibitor such as crizotinib, cabozantinib, tepotinib, and capmatinib. Therefore, the implementation of high-quality and sensitive methods for the detection of the various *MET* aberrations is essential.

**Abstract:**

MET tyrosine kinase receptor pathway activation has become an important actionable target in solid tumors. Aberrations in the *MET* proto-oncogene, including MET overexpression, the activation of *MET* mutations, *MET* mutations that lead to *MET* exon 14 skipping, *MET* gene amplifications, and *MET* fusions, are known to be primary and secondary oncogenic drivers in cancer; these aberrations have evolved as predictive biomarkers in clinical diagnostics. Thus, the detection of all known *MET* aberrations in daily clinical care is essential. In this review, current molecular technologies for the detection of the different *MET* aberrations are highlighted, including the benefits and drawbacks. In the future, another focus will be on the standardization of detection technologies for the delivery of reliable, quick, and affordable tests in clinical molecular diagnostics.

## 1. Introduction

The *MET* gene (MET proto-oncogene, receptor tyrosine kinase), which consists of 21 exons separated by 20 introns, is located on chromosome 7q21-31 and encodes the MET receptor tyrosine kinase (190 kDa). Together with its ligand, hepatocyte growth factor (HGF), MET plays an important role in tumor proliferation, angiogenesis, and migration [1,2]. A variety of *MET* aberrations that lead to the dysregulation of the *MET* oncogene and thus the activation of various signaling pathways such as MAPK, PI3K-AKT, and JAK-STAT have already been described. These include MET overexpression, the activation of *MET* mutations comprising *MET* exon 14 skipping mutations, *MET* gene amplifications, and *MET* fusions [3,4]. Patients with these types of aberrations can be treated by using an inhibitor targeting either *MET*, such as capmatinib and tepotinib, or by using multikinase inhibitors, such as crizotinib and cabozantinib [5]. Capmatinib (Tabrecta^®^, Novartis Pharma GmbH, Basel, Switzerland) and tepotinib (Tepmetko^®^, Merck KGaA, Darmstadt, Germany) have been approved for the treatment of patients with advanced non-small cell lung carcinoma (NSCLC) and *MET* exon 14 skipping mutation who are undergoing systemic therapy after platinum-based chemotherapy or require treatment using immunotherapy. Thus, the implementation of high-quality and sensitive detection methods is essential for the identification of the various *MET* aberrations.

## 2. MET Receptor

**Figure 1:**
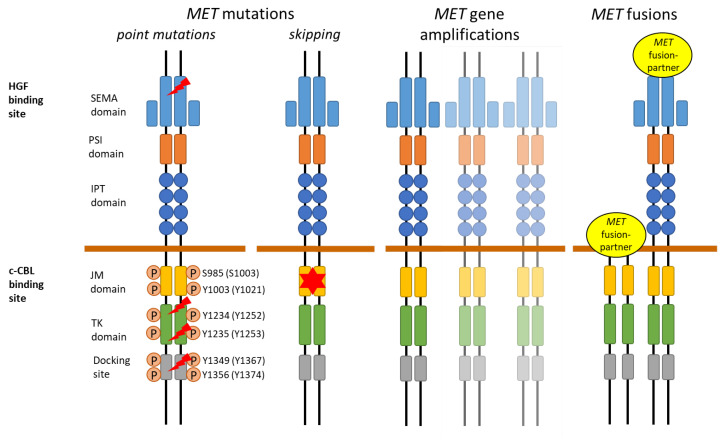
Schematic representation of *MET* aberrations in the MET receptor.

The MET receptor functions as a disulfide-linked heterodimer tyrosine kinase receptor and is composed of an extracellular, transmembrane, and intracellular domain. The extracellular domain is the binding site of its ligand, HGF, and consists of the semaphorin (SEMA), plexin semaphorin integrin (PSI), and immunoglobulin plexin transcription factor (IPT) domains. The intracellular domain consists of the juxtamembrane (JM) domain with the E3 ubiquitin ligase casitas B-lineage lymphoma (c-CBL) binding site, tyrosine kinase (TK) domain, and C-terminal multifunctional docking site (Figure 1) [2,6,7,8]. Binding of the ligand HGF to the SEMA domain on the extracellular portion of the MET receptor induces MET homodimerization and the subsequent autophosphorylation of the tyrosine residues at codon Y1234 and Y1235 (NM_000245 (Y1252 and Y1253 NM_0001127500)) in the intracellular TK domain, thus leading to the activation of the kinase domain. This is followed by the phosphorylation of Y1349 and Y1356 (NM_000245 (Y1367 and Y1374 NM_0001127500)) in the multifunctional docking site, which opens and forms a docking site for intracellular adaptors that recruit SRC (SRC proto-oncogene, non-receptor tyrosine kinase) adapter protein; additionally, the subsequent activation of several downstream pathways occurs, such as the PI3K/AKT pathway, RAS mitogen activated protein kinase (MAPK) cascade, signal transducer and activator of transcription (STAT), and NF-κB pathway [4]. These signaling pathways play an important role in proliferation, organogenesis, liver regeneration, embryogenesis, wound healing, and cell motility [6,7,9].

A variety of *MET* aberrations have been described, including *MET* mutations, *MET* gene amplifications, and *MET* fusions. *MET* point mutations (lightning) can occur in the tyrosine kinase (TK) domain, in the Sema domain, and at the multifunctional docking site.

The main amino acid residues (transcript NM_000245 and NM_0001127500) involved in MET regulation through phosphorylation (P) are depicted. *MET* exon 14 skipping mutations (star) are located in the juxtamembrane (JM) domain, which contains the E3 ubiquitin ligase casitas B-lineage lymphoma (c-CBL) binding site. *MET* fusions are very heterogeneous and can result in different fusion proteins.

## 3. MET Aberrations in Lung Cancer

### 3.1. MET Overexpression

MET overexpression was discovered to be one of the first mechanisms of dysregulation of MET; since its discovery, it has been detected in a variety of cancers [10,11,12]. MET overexpression increases ligand-independent phosphorylation and activation of signaling pathways, and it has been linked to metastases, enhanced tumor invasion, and poor survival [13]. In non-small cell lung cancers (NSCLC), MET overexpression has been found with a varying frequency of 35–72% through immunohistochemistry (IHC) [14,15,16].

Several MET IHC antibodies have been developed, including monoclonal and polyclonal antibodies and antibodies against phosphorylated MET [17,18,19,20]. Most commonly, the anti-c-MET (SP44) rabbit monoclonal primary antibody (Ventana Medical Systems Inc.) is used; however, comparative studies of the different antibodies are still missing. IHC slides are evaluated by pathologists, and MET protein expression is semi-quantitatively measured (Table 1). At present, a variety of MET IHC scoring systems and cutoff values have been published. Mostly, staining intensities are classified as negative (0), weak (1+), moderate (2+), and strong (3+), and a staining intensity of 2+ in at least 50% of tumor cells is classified as MET overexpression [15,17].

Studies that use MET protein expression as a biomarker for MET-targeted therapies with monoclonal antibodies and MET tyrosine kinase inhibitors have not been successful up to this point. This poses the question of whether the cutoff values for patient enrollment have been chosen sufficiently [17,21,22] or whether MET overexpression determined by IHC may have a low predictive value for MET activation and ET tumor dependency [23] in contrast to, for example, ALK overexpression in *ALK* fusion-positive NSCLCs [20]. This is supported by growing evidence that MET IHC cannot be used as a screening tool for MET activation by *MET* exon 14 skipping mutations or *MET* gene amplifications in NSCLC [15]. One study revealed that only 3% of MET IHC positive samples had *MET* exon 14 skipping mutations, and only 1% showed a *MET* gene amplification [15]. Another study showed an only 16.1% concordance between IHC and *MET* DNA alterations (*MET* exon 14 skipping mutations and *MET* gene amplifications) [24]. Thus, MET overexpression as a primary biomarker and oncogenic driver remains unclear and thus has not reached clinical use.

**Table 1:** Detection techniques for *MET* aberrations.

MET Aberration	Detection Technique	Tested Material	Evaluation Criteria	Advantages	Disadvantages
MET overexpression [14,15,16,17,18,19,20,21,22,23]	IHC antibodies	FFPE slide	Semi-quantitative score 0–3+	Technique widely used and available, fast and cheap	Observer-dependent, tissue sectioning artefacts, new FFPE slide for every analysis, no consensus on scoring system and cutoff
*MET* exon 14 skipping [25,26,27,28,29,30,31,32,33,34,35,36,37,38,39]	RNA NGS (amplicon-, AMP-, or hybridization-based)	RNA from FFPE or fresh frozen material	Mutation, coverage, MAF, fusion product of exon 13 and 15	Sensitive, reliable, direct detection of alternative splicing, multiplexing	RNA degradation, underlying mutation cannot be determined
	RT-PCR	RNA from FFPE or fresh frozen material	Fusion product of exon 13 and 15	Sensitive, reliable, direct detection of alternative splicing, fast turnaround time, widely used and available	RNA degradation, underlying mutation cannot be determined, targeted mutations only
*MET* exon 14 skipping mutations and point mutations [33,35,37,40,41,42,43,44]	DNA NGS (amplicon- or hybridization-based)	DNA from FFPE, fresh frozen material, or liquid biopsy	Mutation, coverage, VAF	Sensitive, reliable, detection of exact mutation, multiplexing	No assessment of splicing effect
	Sanger sequencing	DNA from FFPE, fresh frozen material, or liquid biopsy	Mutation, VAF	Detection of exact mutation, fast turnaround time, widely used and available	Sensitivity, single assay for each target, no assessment of splicing effect
*MET* amplifications [5,41,45,46,47,48,49,50,51,52,53,54,55,56,57,58,59,60,61]	FISH	FFPE slides	*MET* GCN, *MET*/CEN7 ratio	Technique widely used and available, detection of focal amplification, polysomy, and chromosome duplications	Observer-dependent, tissue sectioning artefacts, new FFPE slide for every analysis, no consensus on scoring system and cutoff
	DNA NGS (amplicon- or hybridization-based)	DNA from FFPE, fresh frozen material, or liquid biopsy	Mutation, coverage, VAF	Sensitive, DNA from FFPE easily accessible, multiplexing	High number of false negatives, no standardized cutoff or bioinformatics, no morphological correlation
	Other DNA-based technologies (ddPCR, NanoString nCounter technology)	DNA from FFPE, fresh frozen material, or liquid biopsy	Expression, GCN	DNA from FFPE easily accessible	High number of false negatives, no morphological correlation, no standardized cutoff, large amounts of DNA needed
*MET* fusions [35,39,62,63,64,65,66,67,68,69,70,71]	RNA NGS (AMP- or hybridization-based)	RNA from FFPE or fresh frozen material	Fusionreads, 3′-5′ imbalance	Sensitive, reliable detection of known and novel fusion partners, multiplexing	RNA degradation
	DNA NGS (Hybridization-based)	DNA from FFPE	Fusionreads, 3′-5′ imbalance, coverage,	DNA from FFPE easily accessible, detection of known and novel fusion partners if region is covered, multiplexing	False negative results, novel fusions are problematic due to location of fusion break point
	FISH	FFPE slides	n.a. break-apart events	Technique widely used and available	No standardized assay available, observer-dependent, tissue sectioning artefacts, new FFPE slide for every analysis
	RT-PCR	RNA from FFPE	Fusion product	Technique widely used and available	No standardized assay available, only for known fusion partners, RNA degradation

IHC: immunohistochemistry; FISH: fluorescence in situ hybridization; FFPE: formalin-fixed paraffin-embedded; RT-PCR: quantitative real-time polymerase chain reaction; ddPCR: digital droplet PCR; n.a.: not available; GCN: gene copy number; CEN7: centromere of chromosome 7; AMP: anchored multiplex polymerase chain reaction; NGS: next-generation sequencing; VAF: variant allele frequency.

### 3.2. MET Mutations

#### 3.2.1. *MET* Exon 14 Skipping

*MET* exon 14 skipping mutations occur in 3–4% of NSCLC and are very heterogeneous [3]. *MET* exon 14 skipping mutations gained importance when Frampton et al. and Paik et al. first reported large studies that featured patients with stage IV lung adenocarcinomas harboring a variety of *MET* exon 14 splice variants, which resulted in *MET* exon 14 skipping and showed clinical sensitivity towards MET inhibitors [25,27]. To date, over 100 different mutations that can lead to *MET* exon 14 skipping have been described [26]. Mutations leading to *MET* exon 14 skipping interfere with the normal regulation of MET transcription. These include numerous point mutations, insertions, and deletions disrupting the splice acceptor at the branch point or the polypyrimidine tract site in exon 14, the 5′ end of exon 14, or the splice donor site at the 3′ end [28]. Furthermore, silent mutations in the splice sites can lead to *MET* exon 14 skipping. As a consequence, the spliceosome skips transcribing exon 14, leading to the loss of the entire exon and thus the JM domain encompassing 141 base pairs with the c-CBL binding site (Y1003 (NM_000245); Y1021 (NM_0001127500)). Without the c-CBL binding site, ubiquitination by CBL and the subsequent lysosomal degradation of MET is impaired; thus, its downstream signaling pathway is constitutively activated [72]. Additionally, the JM domain contains a second phosphorylation site at codon S985 NM_000245 (S1003 NM_0001127500). Phosphorylation of S985 negatively regulates kinase activity [7].

*MET* skipping mutations are mutually exclusive with mutations in *EGFR*, *KRAS*, and *ERBB2* and fusions in *ALK*, *RET*, and *ROS* [25,27,28,73,74]. Some of the previously reported *MET* mutations seem to represent SNPs; thus, their clinical importance is highly questionable [74]. This problem especially arises when two different mRNA transcripts are frequently used in the literature and when the mutations are reported without the corresponding transcript (transcript NM_000245 and transcript NM_0001127500). For example, in transcript NM_0001127500, a rare polymorphism at codon 1010 (T1010I) is reported; however, in transcript NM_000245, the splice site of exon 14 is located at codon 1010, which can lead to a misconception of the detected mutation in the clinic. The same has been reported with two different *MET* resistance mutations, which are identical variants reported on different transcripts [75]. Thus, mutations should always be reported with the utilized mRNA transcript number [76].

There are several methods available for the detection of *MET* exon 14 skipping mutations (Table 1). They can be either detected on the DNA or RNA level. Increasingly, for the detection of *MET* exon 14 skipping mutations, amplicon- or hybridization-based next-generation sequencing (NGS) methods are used. Although single gene analyzes such as Sanger sequencing or quantitative real-time polymerase chain reaction (RT-PCR) can detect these aberrations, they are used less often and are considered impractical due to the large number of biomarkers that have to be tested simultaneously, especially in lung cancer, and at the same time the low availability of material. The following paragraphs highlight the different methods available.

##### Parallel Sequencing (NGS) Multigene Assays

DNA-based NGS assays analyze the DNA variant that underlies the skipping of exon 14 on the RNA level. For this, *MET* exon 14 as well as the intronic regions up- and downstream of exon 14 have to be covered by NGS assays sufficiently, as *MET* exon 14 skipping alterations are very heterogenous and deletions can reach far into the intronic sites of exon 14 [25]. At the DNA level, the exact mutation can be specified according to the human genome variation society (HGVS) nomenclature, which is not possible on RNA level. However, the splicing effect cannot be directly assessed at the DNA level. To verify the splicing effect, a confirmatory RNA-based assay is necessary or the effect should be substantiated by the literature [77].

There are two main types of NGS assays, amplicon-based and hybridization-based NGS [29,33,35,37,43]. Either custom panels or commercially available panels can be used. However, commercially available targeted as well as whole exome sequencing panels sometimes lack the intronic regions around *MET* exon 14 and are unable to detect all relevant *MET* exon 14 skipping mutations. Amplicon-based assays use a defined set of primers for the enrichment of the target regions via multiplex PCR followed by library preparation and sequencing. The advantage of an amplicon-based approach is the faster turn-around time, demand of smaller DNA amounts, and ability to use even chemically modified and fragmented DNA derived from formalin-fixed and paraffin-embedded (FFPE) tissue [34]. The disadvantages are the limitation of the total target size, possible allele dropout and thus false-negative results if either the mutation is localized within the primer binding site, or the occurrence of primer mismatches, especially in repetitive sequences [77]. Studies have shown that *MET* exon 14 skipping alterations can be missed by amplicon-based sequencing if the assay is not optimized for this purpose [31,36]. When using hybridization-based panels, DNA is initially sheared. During library preparation, target-specific biotinylated capture probes are hybridized to target regions, and the probes are enriched by streptavidin beads before sequencing [29,33,37]. The advantages of this method are that it circumvents allelic dropout and that duplicate sequences can be removed. The disadvantages are that larger amounts of DNA are needed and that the data analysis is highly complex [29,31,33,37]. Furthermore, some assays have poor intronic coverage and off-target sequencing reads reduce the sequencing coverage. In general, hybrid-capture assays often fail to detect larger deletions if the bioinformatic analysis does not enable their detection [77].

In contrast to DNA-based assays, RNA-based assays for the detection of *MET* exon 14 skipping mutations permit the direct detection of alternative splicing of *MET* exon 14, resulting in a fusion of exons 13 and 15. This method’s limitation is that the underlying mutation cannot be determined. However, only the splicing effect is clinically relevant and qualifies for targeted therapy [3,31]. For RNA-based assays, amplicon-based and hybridization-based panels can also be used. Additionally, an anchored multiplex polymerase chain reaction (AMP) approach can be utilized, which has shown promising results [39]. This technology uses a single-primer extension approach without predefined amplicon sizes. With this technology, fusions and splice variants can be detected without knowledge of the fusion partner, as only one target primer is included [4]. RNA-based assays, however, are highly dependent on the RNA integrity. RNA quality should be closely monitored, especially when using FFPE material. Ideally both DNA- and RNA-based approaches should be simultaneously used. However, as many laboratories perform the DNA extraction first followed by the DNA-based NGS analysis, often no material is left for RNA-based sequencing, especially when using lung cancer biopsies [31,77]. Thus, ideally a combined DNA and RNA extraction from the same tissue slides should be performed.

##### Single Gene Analyzes

Sanger sequencing can also be used for the detection of *MET* exon 14 skipping mutations. It is material-consuming, as a single DNA fragment is sequenced at the time and shows low sensitivity because of it only being able to detect mutations with an allele frequency above 20%. Thus, a higher tumor cell content is needed than for other sequencing technologies. On the other hand, it is easy to implement and allows for the detection of previously unknown alterations if the region is covered. At the RNA level, the RT-PCR technique is widely used in laboratories for the detection and confirmation of *MET* exon 14 skipping variants, as this method is a cost-effective and fast approach to test FFPE material with high sensitivity [30,38]. However, these assays fail to detect additional mutations and should only be used as a pre-screening or confirmatory tool. Newly developed multigene RT-PCR assays from Biocartis NV (Mechelen, Belgium), Diatech Pharmacogenetics S.R.L. (Jesi AN, Italy), or AmoyDx (Xiamen, China) overcome this issue and allow for the detection of a variety of gene fusions and splice variants at the same time in an easy-to-use and time-sensitive manner [32].

#### 3.2.2. Other MET Mutations

In addition to *MET* exon 14 skipping mutations, activating point mutations in the TK, JM, and extracellular domains have been reported in cancer, leading to ligand-independent receptor phosphorylation and signaling (Figure 1 and Table 1) [78,79,80]. A variety of these mutations, including H1094Y/R/L (NM_000245; H1112, NM_0001127500) and D1228H/N (NM_000245; D1246, NM_0001127500), were first described in hereditary papillary renal cell carcinoma (HPRCC) [44] and were later also found in sporadic papillary renal cell carcinoma (PRCC) with up to a 15% frequency [40,42].

In NSCLC, *MET* mutations in the TK domain are rare and mainly emerge as an acquired resistance mechanism to MET tyrosine kinase inhibitors or as a resistance mechanism to combinational therapy with EGFR and MET TKI in *MET* exon 14 skipping positive patients or *EGFR*-mutant and *MET* gene amplification positive patients. Mutations such as Y1230C (NM_000245; Y1248C, NM_0001127500), Y1230H (NM_000245; Y1248H, NM_0001127500), D1228H (NM_000245; D1246H, NM_0001127500), and D1228N (NM_000245; D1246N, NM_0001127500) were found to mediate resistance by disrupting the drug binding site of crizotinib [3,81].

*MET* mutations in the SEMA domain and extracellular compartment have also been reported to possibly affect ligand binding. However, the functional significance and relevance of these mutations is still unknown [13]. Thus, it remains important to evaluate the functional consequences of other *MET* mutations and their clinical implications.

### 3.3. MET Gene Amplification and Gene Copy Number Alterations

*MET* gene amplifications and copy number alterations have been reported in 1–6% of NSCLC [61]. Therapy approaches for NSCLC with *MET* gene amplifications, which both occur as the primary driver aberration and as the resistance mechanism to other kinase inhibitors, have currently been evaluated in numerous studies. Studies have shown that MET inhibitors are particularly effective in highly amplified (high level) tumors with a gene copy number [GCN] ≥ 10, were *MET* gene amplification acts as an oncogenic driver [5,55,61]. Thus, patient selection and the exact analysis of the *MET* gene amplification status is crucial.

*MET* gene amplifications and copy number alterations arise from the focal or regional amplification of the *MET* genomic region or from polysomy (Figure 1). In cases with focal amplification, *MET* GCN gains occur without chromosome 7 duplication, whereas *MET* GCN gains due to polysomy arise from the duplication of parts or the entire chromosome 7; thus, multiple parts of chromosome 7 are present [3,47].

*MET* gene amplifications can be assessed using a variety of methodologies, which determine either the average *MET* GCN and/or the ratio to the centromeric region of chromosome 7 (Table 1). However, the cutoff point for setting MET positivity is still very variable. Different clinical studies have used a variety of thresholds for the definition of amplifications, from amplification positive only to defining an exact *MET* GCN [22,46,49,56,61]. Depending on the method used, thresholds are set at different levels, which causes problems in the interpretation of the potential of *MET* GCN as true biomarker [47].

Additionally, true gene amplifications without chromosome 7 duplication are more likely to lead to oncogene addiction [3,61].

#### 3.3.1. Fluorescence In Situ Hybridization (FISH)

*MET* gene amplifications and *MET* GCN can be detected using various methods. The gold standard for the detection of *MET* gene amplifications and the most accurate detection method is still fluorescence in situ hybridization (FISH), which is currently still superior to DNA-based methods and mainly used in clinical trials. Bicolor FISH probes label both the *MET* gene and the centromere of chromosome 7 (CEN7). The number of signals identified in a nucleus represent the number of copies present. The signals in a predefined number of cell nuclei are counted and scored based on evaluation criteria such as the *MET* gene/CEN7 ratio and/or the average *MET* GCN. According to the resulting score, cases are divided into different groups [48,50,53,54,58]. Various evaluation scores have been published so far. Thus far, publications have defined *MET* gene amplification by GCN only, either as 5 or more copies per cell [48], or as *MET* GCN ≥ 6, ≥10, or 15 [54,55]. Other studies have also included the number of chromosomes present by calculating *MET*/CEN7 ratio; thus, true amplification can be distinguished from polysomy. A *MET*/CEN7 ratio of ≥2.0 is commonly defined as *MET* gene amplification [50,53,58]. Other studies have categorized the degree of amplification into low, intermediate, high-level, and top-level amplified cases. A top-level amplification was classified as an average *MET* GCN per cell of ≥10. a high-level amplification was defined in tumors with a *MET*/CEN7 ratio ≥2.0 or an average *MET* GCN per cell of ≥6. An intermediate level of GCN gain means that ≥50% of cells contain ≥5 *MET* signals. A low level of GCN gain was defined as ≥40% of tumor cells showing ≥4 *MET* signals [55,58].

The FISH technique is especially useful in cases with low tumor cell content, tumor heterogeneity, and focal amplifications, as FISH is performed on slides and evaluated under the microscope [52,58,60]. However, in situ-based approaches like FISH are also thereby hampered. The evaluation is observer-dependent, and tissue sectioning artefacts can impact the analysis. Furthermore, a new slide of material must be used for each additional parameter that is tested by FISH, which can be problematic when using small biopsies [52,60].

#### 3.3.2. DNA-Based Methods

Another option for the detection of *MET* gene amplifications are a variety of DNA-based methods that work with extracted nucleic acids, such as digital droplet PCR (ddPCR), next-generation sequencing (NGS), or the NanoString nCounter technology. The detection of *MET* gene amplifications by GCN changes using DNA-based methods is still under evaluation. These methods allow for an easier quantification of GCN in comparison to FISH but do not allow for morphological correlation. At present, the performance of NGS-based assays for the detection of *MET* gene amplification have been characterized the best. Data for the other DNA-based methods mentioned above are very limited [52]. Studies that have compared NGS and FISH assays showed low consistency between both methods. Currently, only high-level and top-level amplified samples with GCN ≥ 10 and negative samples determined by FISH can be reliably detected by an NGS analysis [3,52,57,59,60]. Comparative studies have further shown that *MET* gene amplifications can be missed by NGS assays due to a variety of reasons. On the one hand, the tumor material itself can pose problems in the evaluation due to low tumor purity (inclusion of normal, necrotic, and inflammatory cells), low tumor cell content, the overall amount of material present, the FFPE DNA quality, tumor heterogeneity, or focal amplifications and polysomy [52,57,59,60]. On the other hand, analyzing large genomic alterations can be very challenging and can create computational challenges, such as call accuracy and noise reduction. Additionally, a defined set of normal samples or standardized set of controls and the tumor cell content of the samples have to be used for bioinformatic analyses [41,45,51].

In molecular diagnostics, amplicon-based as well as hybridization-based NGS assays are used for *MET* GCN detection. Hybridization-based NGS assays can assess *MET* GCN variations more accurately than amplicon-based NGS assays, as the sequencing bias is reduced, duplicate reads can be filtered out, and the true mean coverage used for GCN determination is less affected by DNA quality, tumor complexity such as tumor purity, and heterogeneity [3,60,78,79,80]. However, to date, there are no methodologically or clinically defined cutoffs for the definition of *MET* positivity when utilizing NGS assay, nor is there an accepted standard or general consensus regarding the protocols and bioinformatics used, which inevitably leads to discordant results across studies.

### 3.4. MET Fusions

*MET* gene fusions are rare oncogenic driver alterations in a variety of cancers, including hepatocellular carcinoma, gastric carcinoma, sarcoma, and NSCLC. Only in glioblastomas, *MET* fusions are described in 12% of cases [3]. The frequency of *MET* fusions in NSCLC is < 0.5%, and they are found to be mutually exclusive with other oncogenic drivers [66]. The first *MET* fusion identified in lung cancer was the *TPR*-*MET* fusion [82]. Since then, several fusion partners have been characterized, such as *KIF5B*, *CLIP2*, *TFG*, *STARD3NL*, *ATXN7L1*, *PTPRZ*, and *CD74* [63,64,66,67,71]. *MET* fusions occur through inter- or intra-chromosomal rearrangement and mostly include the kinase domain on exon 15 and downstream, resulting in ligand-independent constitutive MET activation (Figure 1) [65,66,67,68]. The *TPR*-*MET* fusion, however, does not include exon 14 of *MET* and can show the same oncogenic behavior as NSCLCs with *MET* exon 14 skipping [70]. Fusions such as *KIF5B*-*MET* and *PTPRZ*-*MET* that include exon 14 appear to be less oncogenic than the *TRP*-*MET* fusion [65]. In the *PTPRZ*-*MET* fusion protein, the *MET* gene is present in the full length, including in the dimerization domain in exon 2, resulting in MET overexpression and increased activation [65]. Therefore, the knowledge of the exact fusion break point seems to be important for the success of MET TKIs. Currently, clinical trials are evaluating the efficiency of MET TKIs in *MET* fusion-positive cancers.

For the detection of *MET* fusions, an RNA-based NGS approach that uses either AMP- or hybridization-based technologies is the first choice. In this way, both unknown fusion partners and the involved exons can be determined [62]. Alternatively, FISH, RT-PCR, and DNA-based NGS techniques can be used. However, an RNA-based NGS panel analysis is the most sensitive approach for such rare and novel events, as all relevant fusions and *MET* exon 14 skipping mutations can be detected in just one assay, thus making FISH and RT-PCR inadequate detection tools. Additionally, intrachromosomal rearrangements may lead to false negative FISH results, as the distance between the 5′ and 3′ probes are too short [35,39,62,71]. DNA-based hybrid-capture NGS approaches that can detect both mutations and fusions have proven to be unreliable in the past and often lead to false negative results for fusion detection, especially in cases of novel fusions. This is due to the localization of fusion breakpoints in large intronic regions with repetitive sequences, which are difficult to cover using capture probes [35,54,69].

## 4. Conclusions

In recent years, the large, growing number of detected *MET* alterations in NSCLC and other carcinomas as well as the better understanding of the diverse biology driving MET dysregulation in cancer has shown the important role of this kinase for targeted therapy approaches.

Particularly, since the FDA and EMA approval of MET inhibitors for NSCLCs with *MET* exon 14 skipping mutations, testing for all *MET* alterations, e.g., MET expression, *MET* mutations, *MET* gene amplifications, and *MET* fusions, should be routine standard of care for patients with NSCLC. However, there is still the need for the further development of quality assured and sensitive molecular detection methods, especially under the new In Vitro Diagnostics Regulation (IVDR) and when it comes to the detection of *MET* gene amplifications, as these are still widely analyzed reliably by only FISH while the cutoffs for other technologies are lacking. Additionally, inconsistent nomenclature of somatic variants and the different transcripts used in the literature are still of concern, as this can lead to a misconception of the detected mutations in the clinic and thus therapeutic failure. As a final note, quality assured and sensitive molecular detection methods are especially important in Europe, as laboratories are free to choose the diagnostic method used for the detection of the different *MET* alterations.

**Figure 2:**
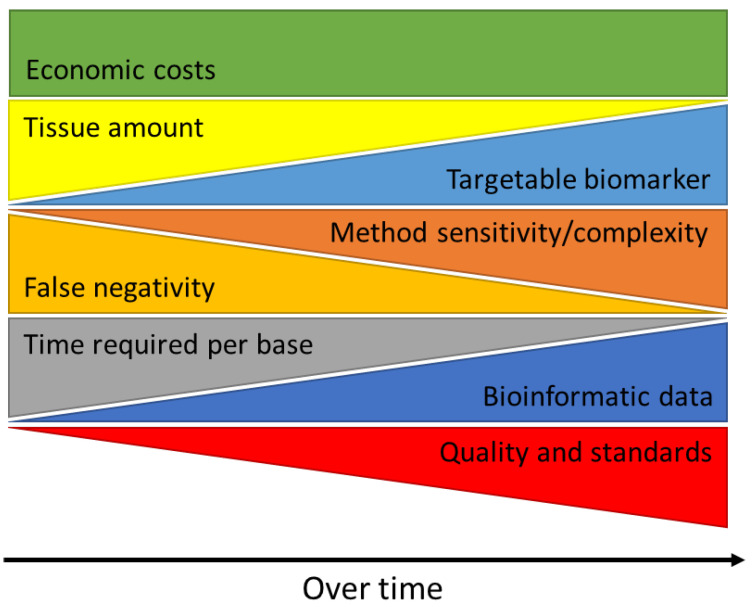
Clinical utility of the analysis of tumor material in molecular pathology diagnostics over time.

In the future, the number of targetable biomarkers will increase more and more, and the amount of tissue, effort, and time required to complete complex diagnostic tests will become even more limiting. As molecular targets and therapeutic approaches are continuously changing, the ongoing development and implementation of high-quality molecular testing, and the continuous adaption to the latest findings in cancer research will become increasingly important while limiting economic costs at the same time (Figure 2).
